# Multifocal bone tuberculosis simulating metastasis

**DOI:** 10.1002/ccr3.4536

**Published:** 2021-07-23

**Authors:** Imen Chabchoub, Raida Ben Salah, Wien Feki, Zeineb Mnif, Zouhir Bahloul

**Affiliations:** ^1^ Department of Internal Medicine Hedi Chaker Hospital Sfax Tunisia; ^2^ Department of Radiology Hedi chaker Hospital Sfax Tunisia

**Keywords:** Bone Tuberculosis, infections, metastasis

## Abstract

Multiple skeletal tuberculosis can be the first sign of tuberculosis. In such cases, physicians should consider tuberculosis diagnosis and take biopsies for anatomopathological evidence to make the correct diagnosis.

## Graphical abstract

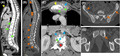

## 1 INTRODUCTION

A 22‐year‐old woman was admitted to our hospital with several bone lesions suggestive of skeletal metastases. In such cases, clinicians should consider the possibility of tuberculosis (TB) as a probable diagnosis and obtain samples via biopsy for pathological confirmation. In our case, anti‐tuberculosis drug treatment was successful in curing the lesions.

Tuberculosis (TB) is a major public health concern worldwide.^1^ While most of TB infections affects the lungs, it can also affect other sites, giving rise to a form known as extrapulmonary EPTB.^2^ The most common anatomic locations of extrapulmonary TB are lymph nodes, pleura, bone and joints, urogenital tract, and meninges.^3^ Multifocal Skeletal TB is rare, accounting for 5% of all the TB cases.^4^

The diagnosis of bone tuberculosis is a genuine challenge for the clinician because the clinical samples obtained are from relatively inaccessible sites that may be paucibacillary, thus compromising the accuracy of diagnostics tests by decreasing their sensitivity.

We hereby present a case of multifocal skeletal tuberculosis simulating metastasis.

## 2 CLINICAL CASE

A 22‐year‐old undergraduate student suffering from a painful sternal swelling was admitted to our hospital. She has also been complaining of significant weight loss and intermittent fever in the past six months.

There was no history of Koch's or Koch's contagion. General examination of the patient did not reveal any significant abnormality except for localized tenderness over the sternum. No abnormal signs were found in the neurologic examination. Laboratory results were as follows: Routine blood test: WBC 6.1 × 109/L, HGB 94 g/L, PLT 338 × 109/L, and serum albumin level 33.2 g/L. Plasma protein electrophoresis and immunofixation electrophoresis were normal. The erythrocyte sedimentation rate (ESR) and Creactive protein (CRP) level were 73mm/hr (and 110 mg/L (normal value 0–8 mg/L), respectively. Serum tumor markers, including AFP, CEA, CA199, CA 125, and CA 15–3, were all within normal ranges. Hepatitis B surface antigen (HBsAg) and HIV were negative. Tuberculin skin test (TST) and Interferon gamma by T‐SPOT were negative.

**FIGURE 1:**
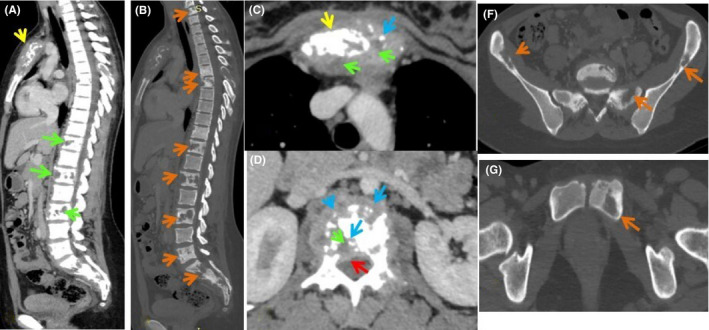
Thoraco‐abdominopelvic CT after contrast injection: Sagittal (A, B), axial (C, D, F, G), parenchymal (A, C, D) and bone (B) windows (F, G): Multiple osteolytic lesions surrounded by peri‐lesional osteo‐condensation of the cervico‐dorso‐lumbo‐sacral spine, iliac wings and left pubis (orange arrows) with rupture of the cortical bone and bone sequestration by location (light blue arrows) real masses in the sternum (yellow arrows) and sacrum (dark blue arrow). Evidence of multiple abscesses intra‐somatic and sternal (green arrows). It associates an epiduritis at L1 level (red arrow)

A computed tomography scan (Figure 1) revealed multiple osteolytic lesions surrounded by peri‐lesional condensation of the spine, iliac wings, and left pubis with rupture of the cortical bone and bone sequestration in some places realizing a genuine mass in the sternum. There are multiple abscesses in the sternum, spine, and extension of lesions in the epidural space at L1 level. Enhanced chest computed tomography (CT) studies did not show any parenchymal lung damage or lymphadenopathy.

A body scan revealed multiple osteolytic lesions with intense FDG uptake, supporting the diagnosis of skeletal metastases.

Two fine needle aspiration biopsies of the sternal lesion showed that both bones and cartilages had a small amount of fibrous tissue attached to them.

Surgical biopsy obtained from the iliac lesion identified epithelioid cell nests, multinuclear giant cell, and cheesy necrosis, which were typical manifestations of tuberculosis. The diagnosis of multifocal skeletal tuberculosis was confirmed. After standard anti‐TB drug treatment for 12 months, the patient's discomfort was remarkably relieved.

## 3 DISCUSSION

Tuberculosis with multifocal bone involvement is extremely rare in immunocompetent patients and in those with normal pulmonary findings, amounting to 5% of all the TB cases.^4^

However, multiple non‐contiguous vertebral involvements accounts for only 1.1–16% of skeletal TB.^5^ The diagnosis of bone tuberculosis is a genuine challenge for clinicians.

For the best interest of patients, the diagnosis of malignancy is to be suspected in the first place.

Firstly, our patient was immunocompetent without any history of contagion or pulmonary complaints. Secondly, unusual radiological images were observed, which were more compatible with a hematological malignancy or metastatic disease rather than an infectious disease. Thirdly, a negative Interferon gamma result was also a contributing factor to the delayed diagnosis, and finally, results from two biopsies were non‐contributive. Only, a surgical biopsy did confirm the diagnosis of tuberculosis by identifying caseous granulomas.

Conventional acid‐fast bacilli smears have low sensitivity, and it requires a long time for M. tuberculosis to become evident during culture.

In general, the possibility of detecting Mycobacterium may be less than 50% in musculoskeletal tuberculosis.^6^ As a result, the diagnosis of extrapulmonary tuberculosis (EPTB) mostly depends on histological evidence.

Numerousbiopsies must be performed and more time and attention ought to be given to microscopy to improve the rate of correct diagnosis of skeletal tuberculosis.

For histopathological diagnosis, presence of granulomas and caseation have been commonly used to define a positive test. Pathology findings of a greater suppurative response and a lesser number of well‐formed granulomas may be a result of a loss of host immune function.^7^

Tuberculin skin test (TST) and IFN‐γ releasing assay may be of aid for diagnosing EPTB, but it has a limited diagnostic value.

A negative IFN‐γ releasing assay as in our case cannot eliminate tuberculosis. Individuals suspected of having tuberculosis showed a sensitivity of 69–83% and specificity of 52–61% for T‐SPOT.^8^ T‐SPOT cannot distinguish latent tuberculosis from active tuberculosis, and it is not overly specific for active tuberculosis.^9^, ^10^

Anti‐TB drug treatment is the mainstay in the management of any form of tuberculosis. However, the duration of the treatment is one of the controversial aspects of the management of EPTB. Although 6months of standard anti‐TB drug treatment is generally considered adequate for most forms of EPTB, longer treatment is suggested for bone and joint TB. In the case of skeletal and joint TB, some guidelines recommend 6‐month drug treatment regimens, which frequently achieve microbiologic and clinical cure.^11^ However, many specialists still prefer a treatment duration of more than 12 months due to the difficulties in both assessing treatment response and defining the response to treatment.^12^ In our case, after standard anti‐TB drug treatment for 12 months, his discomfort was relieved remarkably.

## 4 CONCLUSION

Multifocal skeletal tuberculosis remains rare entity. The diagnosis of bone tuberculosis is a considerable challenge for clinicians. Our patient was immunocompetent without any history of contagion or pulmonary complaints and radiological images were more compatible with a hematological malignancy or metastatic disease. In similar challenging cases, clinicians should mention of the possibility of TB diagnosis and perform the necessary biopsies for pathological proof in order to get the right diagnosis.

## CONFLICT OF INTEREST

The authors declare that they have no competing interests.

## AUTHOR CONTRIBUTIONS

R Ben Salah and I Chabchoub: collected data and information and were the major writers of the present work. W Feki: performed, described, and analyzed the radiography in the present work, and contributed to manuscript writing; Z Bahloul, Z Mnif: contributed to data analysis and manuscript writing;

## ETHICAL STATEMENT

The patient has consented to publication the case, imaging and all data.

## STATEMENT ABOUT DIGITAL PHOTOGRAPHS

The authors declare that all the digital photographs included in this work have not been modified, edited, or adulterated in any way.

## Data Availability

Data sharing is not applicable to this article as no new data were created or analyzed in this study.
